# Sample size calculations based on a difference in medians for positively skewed outcomes in health care studies

**DOI:** 10.1186/s12874-017-0426-1

**Published:** 2017-12-02

**Authors:** Aidan G. O’Keeffe, Gareth Ambler, Julie A. Barber

**Affiliations:** Department of Statistical Science, University College London, Gower St., London, WC1E 6BT UK

**Keywords:** Hypothesis test, Log-transformation, Median, Sample size, Skewness

## Abstract

**Background:**

In healthcare research, outcomes with skewed probability distributions are common. Sample size calculations for such outcomes are typically based on estimates on a transformed scale (e.g. log) which may sometimes be difficult to obtain. In contrast, estimates of median and variance on the untransformed scale are generally easier to pre-specify. The aim of this paper is to describe how to calculate a sample size for a two group comparison of interest based on median and untransformed variance estimates for log-normal outcome data.

**Methods:**

A log-normal distribution for outcome data is assumed and a sample size calculation approach for a two-sample t-test that compares log-transformed outcome data is demonstrated where the change of interest is specified as difference in median values on the untransformed scale. A simulation study is used to compare the method with a non-parametric alternative (Mann-Whitney U test) in a variety of scenarios and the method is applied to a real example in neurosurgery.

**Results:**

The method attained a nominal power value in simulation studies and was favourable in comparison to a Mann-Whitney U test and a two-sample t-test of untransformed outcomes. In addition, the method can be adjusted and used in some situations where the outcome distribution is not strictly log-normal.

**Conclusions:**

We recommend the use of this sample size calculation approach for outcome data that are expected to be positively skewed and where a two group comparison on a log-transformed scale is planned. An advantage of this method over usual calculations based on estimates on the log-transformed scale is that it allows clinical efficacy to be specified as a difference in medians and requires a variance estimate on the untransformed scale. Such estimates are often easier to obtain and more interpretable than those for log-transformed outcomes.

**Electronic supplementary material:**

The online version of this article (doi:10.1186/s12874-017-0426-1) contains supplementary material, which is available to authorized users.

## Background

In most clinical studies, sample size calculations are important at the study design stage [1–3]. A typical objective of such studies is to test for a difference in the distribution of some outcome of interest between two or more groups using a hypothesis test. A sample size calculation helps to ensure that a study has the correct power to reject the null hypothesis, thereby providing conclusive evidence of a true difference between groups, where such evidence exists. Where a study does not have a high level of power, the probability of rejecting the null hypothesis is low and, as a result, evidence in support of a difference between groups may not be detected.

For studies in which the outcome of interest has a continuous distribution, a two-sample t-test is often used to test the null hypothesis that the mean outcome is the same for two groups. Use of the two-sample t-test relies on an underlying assumption that this outcome is normally distributed. The required sample size is then calculated on the basis of a pre-specified minimum clinically significant difference in means between groups and an estimate for the variance of the outcome (or, equivalently, a standardised mean difference), together with a desired hypothesis test power and significance level. However, many health care outcomes are not normally distributed and instead have skewed distributions. For example: quality-of-life measures [4], tumour size or features in cancer patients [5, 6] and time outcomes [7, 8], amongst many others. For such data, non-parametric tests might be considered (for example, a Mann-Whitney U test) although such tests may be affected by a reduced power and can require a substantial inflation of the sample size [9, 10]. Alternatively, outcomes may be transformed to obtain a normal distribution and enable the use of a two sample t-test. For positively skewed outcome data, a common transformation is the natural logarithm of the outcome [11] with a t-test then used to test the null hypothesis that mean values on the log-transformed scale are equal. In this case, standard sample size calculations based on t-tests or Z-tests are performed for the transformed data, using pre-specified means and variances on the log-scale.

A potential problem with taking this approach can be specifying accurate or appropriate values of standardised mean differences (or, equivalently, group means and standard deviations) for the log-transformed outcomes. Often, it may be more natural for health care practitioners or trialists to have knowledge of such values on the untransformed scale, since these typically represent more clinically relevant or meaningful measures. A natural choice for summarising skewed data on the untransformed scale is the median. Unlike the mean, the median is not unduly influenced by extreme values. Estimates of medians are likely to be more readily available and interpretable than the alternative of specifying means on the log-scale. In addition, specifying variances of untransformed values is also likely to be easier for a study’s research team and probably more accurate than the specification of variances on the log-scale.

Here, we describe how a sample size can be calculated for a two group comparison of a log-normal outcome, based on estimates of the median outcome and untransformed standard deviation in each group and evaluate this approach in a variety of settings. The method is relevant for both randomised trials and observational studies where we plan to test the null hypothesis that log-scale means are equal or, equivalently, the null hypothesis that medians on the untransformed scale are equal. The approach can be extended using usual methods to incorporate other complexities such as clustering, unequal allocation or adjustments for confounding.

## Methods

We assume that we have a study for which the outcome of interest is positively skewed and that we wish to perform a hypothesis test that compares outcomes between two independent groups, for example, a parallel-group randomised controlled trial in which outcomes are compared for a placebo group and an active treatment group. Throughout, we define the two groups as groups 1 and 2. Furthermore, for simplicity, sample size calculations are performed assuming that these groups are equally sized. Assuming that each group has size *n*, we denote *T*_*ij*_ as the positively skewed outcome value for individual *i* (*i*∈{1,…,*n*}) in group *j* (*j*∈{1,2}). We assume that, in each group, the primary outcome *T*_*ij*_ has a log-normal distribution. That is

$$T_{ij}\sim\text{log} \mathcal{N}\left(\mu_{j},\sigma^{2}_{j}\right) $$

and hence

$$\log(T_{ij}) \sim \mathcal{N}\left(\mu_{j},\sigma^{2}_{j}\right). $$

To compare groups, the usual null (H_0_) and alternative (H_1_) hypotheses tested would be:

$$\begin{array}{*{20}l} ~&\mathrm{H}_{0}: \mu_{1}=\mu_{2};\\ \text{versus}&~\\ ~&\mathrm{H}_{1}: \mu_{1}\neq\mu_{2}. \end{array} $$

In words, this denotes a test of the null hypothesis that the log-scale means are equal for groups 1 and 2 against a two-sided alternative. For a log-normal distribution, this test is equivalent to a test of the null hypothesis that the medians of the untransformed outcomes are equal for groups 1 and 2.

This test could be performed using a two-sample t-test between groups with the log-transformed outcome values. A standard sample size calculation for such a test would rely on the specification of a minimal clinically relevant difference in mean log-transformed outcome values between groups together with an estimate of the variance of the log-transformed primary outcome values for each group. For many outcomes, health care researchers or clinicians may not be familiar with their outcome on the log-scale and may find it difficult to specify the requested estimates. In contrast, clinicians may have a more precise idea of the approximate median outcome value for each group together with an appreciation of the variance of the untransformed outcome values, perhaps from pilot studies, clinical observation/expertise or results in relevant literature. Alternatively, an estimate of the median for the control group could be specified, together with an anticipated difference in the median values between groups for the other group on conclusion of the study.

Where outcome data have a log-normal distribution, the mean of the log-transformed outcome can be easily calculated as the natural logarithm of the median on the untransformed scale. However, it is more challenging to recover the variance of the log-transformed outcomes and, in many cases, pre-specifying variances of log-transformed outcome data may not be straightforward. We now demonstrate how the sample size calculation for a two group t-test comparison of a log-normal outcome can be obtained using medians and variances specified for each group on the untransformed scale. We define

$$\begin{array}{*{20}l} m_{j} &= \text{~ }\\ &\qquad\text{outcome for group {j};}\\ \phi^{2}_{j} &= \text{~Variance of the untransformed primary}\\ & \qquad\text{outcome for group {j}.} \end{array} $$

It can be shown (see Additional file 1, or [12]) that

$$m_{j} = \exp(\mu_{j}), $$

due to the symmetry of the distribution of log(*T*_*i*_). In other words, for a log-normal random variable, the population-level geometric mean (log-scale mean) is equal to the population median. As a result, on specification of approximate median values of the primary outcome for the two groups, the difference in means on the log-scale is written

1 $$\begin{array}{*{20}l} \tau &= \mu_{1}-\mu_{2}\\  &= \log(m_{1})-\log(m_{2}). \end{array} $$

Here, *τ* corresponds to the minimal clinically important difference in the primary outcome on the log-scale, but we note that this has been constructed using median values of the untransformed primary outcome, which may be easier to pre-specify. The variance of the untransformed outcome for group *j* is $\phi ^{2}_{j}$ and, as mentioned previously, it is likely that the variance of an untransformed outcome is easier and more meaningful to pre-specify than that of transformed outcome variable. It can be shown (see ‘Additional file 1’) that the variance of the log-transformed primary outcome for group *j*, $\sigma _{j}^{2}$, is related to the variance of the corresponding untransformed primary outcome as follows

2 $$ \sigma_{j}^{2} = \log\left(\frac{1}{2}+\sqrt{\frac{1}{4}+\frac{\phi^{2}_{j}}{m_{j}^{2}}} \right).  $$

To compare groups, a two-sample t-test is performed using log-transformed outcome variables. The hypotheses are given by

3 $$\begin{array}{*{20}l} ~&\mathrm{H}_{0}: \mu_{1}=\mu_{2};\\  \text{versus}&~\\  ~&\mathrm{H}_{1}: \mu_{1}\neq\mu_{2}. \end{array} $$

In other words, the test is of the null hypothesis that the log-scale means are equal. We note that *μ*_*j*_= log(*m*_*j*_) (*j*∈{1,2}) and, as such, this test may be used to test the null hypothesis of equal medians on the untransformed scale. Taking the standard sample size calculation formula for a two-sample t-test with equal group sizes [13] and using (1) and (2), the number of patients per group (*n*) is given by

4 $$ \begin{aligned} n &= \frac{\left(\sigma_{1}^{2}+\sigma_{2}^{2}\right)\left(z_{\frac{\alpha}{2}}+z_{\beta}\right)^{2}}{\left(\log(m_{1})-\log(m_{2})\right)^{2}}\\ &= \frac{\left[\log\left(\frac{1}{2}+\sqrt{\frac{1}{4}+\frac{\phi^{2}_{1}}{m_{1}^{2}}}\right)+\log\left(\frac{1}{2}+\sqrt{\frac{1}{4}+\frac{\phi^{2}_{2}}{m_{2}^{2}}}\right)\right]\left(z_{\frac{\alpha}{2}}+z_{\beta}\right)^{2}}{(\log(m_{1})-\log(m_{2}))^{2}}. \end{aligned}  $$

Here, *z*_*ε*_ denotes the value such that $\mathbb {P}(Z>z_{\epsilon }) = \epsilon $ for a standard normal random variable $Z\sim \mathcal {N}(0,1)$. So $z_{\frac {\alpha }{2}}$ and *z*_*β*_ denote quantiles pertaining to a significance level of 100*α* *%* and power of 100(1−*β*)*%*. To summarise, Eq. (4) allows a sample size calculation to be performed easily for a two group comparison of untransformed medians or log-scale means on pre-specification of untransformed medians, *m*_1_ and *m*_2_ and untransformed standard deviations *ϕ*_1_ and *ϕ*_2_.

A common approach taken when conducting sample size calculations for normally distributed outcomes is to assume a common standard deviation for the outcomes in both groups. With the method considered in this paper, an assumption of common standard deviation values for the untransformed outcomes (i.e. *ϕ*_1_=*ϕ*_2_) would still imply that the standard deviations of the transformed outcomes (*σ*_1_ and *σ*_2_ in Eq. (2)) are different, owing to the expected difference between *m*_1_ and *m*_2_. In addition, the formula for the sample size given in Eq. (4) is based on a normal distribution but where the hypothesis test of interest is a two-sample t-test. As a result, for smaller sample sizes, it may be sensible to increase the sample size slightly, in line with other sample size calculation methods for a two-sample t-test.

The method presented in Eq. (4) is not only applicable to situations where a comparison of medians between groups is desired. The relationship between the untransformed median and the log-scale mean for a log-normal distribution also implies that the method is useful for situations in which linear regression models are fitted to log-transformed data and used for inference under an assumption that the log-transformed outcomes are approximately normally distributed. Such models are used frequently in medical statistics.

To explore and evaluate the accuracy of Eq. (4) as a method for sample size calculation, we perform a simulation study in which the power of the hypothesis test can be estimated in a variety of scenarios and compared to other common approaches.

## Simulation study

To perform the simulation study, pre-specified values of untransformed median values (*m*_1_ and *m*_2_) were chosen together with corresponding untransformed standard deviations *ϕ*_1_ and *ϕ*_2_. Using these parameters, and for a chosen power and significance level, a sample size *n* was calculated analytically using the method outlined in “Methods” section, specifically Eq. (4). The aim was to assess whether or not the analytically derived sample size would attain the desired level of power when a two-sample t-test comparing log-transformed outcomes between groups is performed. The null and alternative hypotheses for this test were specified in the previous section (3). In addition, we aimed to compare this test to a Mann-Whitney U test and a two-sample t-test that compared untransformed outcomes between groups. The algorithm for the simulation process was as follows:

1. At random, draw *n* values from the distribution $\log \mathcal {N}\left (\mu _{1},\sigma _{1}^{2}\right)$ and *n* values from the distribution $\log \mathcal {N}\left (\mu _{2},\sigma _{2}^{2}\right)$ where, for *j*∈{1,2}:
  $$\begin{array}{*{20}l} \mu_{j} &= \log(m_{j});\\ \sigma^{2}_{j} &= \log\left(\frac{1}{2}+\sqrt{\frac{1}{4}+\frac{\phi_{j}^{2}}{m_{j}^{2}}}\right). \end{array} $$
  The two sets of drawn values are denoted (*T*_11_,…,*T*_*n*1_)^*T*^ and (*T*_12_,…,*T*_*n*2_)^*T*^ respectively.
2. The corresponding log-transformed values are computed as *Y*_*ij*_= log(*T*_*ij*_), producing two sets of log-transformed outcomes (*Y*_11_,…,*Y*_*n*1_)^*T*^ and (*Y*_12_,…,*Y*_*n*2_)^*T*^. These are compared using a two-sample t-test of the null hypothesis that *μ*_1_=*μ*_2_ (equivalently, *m*_1_=*m*_2_ on the untransformed scale) against a two-sided alternative and assuming a 5% significance level. The outcome of the test is recorded using a binary variable (1 = ‘reject the null hypothesis’, 0 = ‘retain the null hypothesis’). In each case, a two-sample t-test is performed to compare log-transformed outcomes between groups. In addition a Mann-Whitney U test and a two-sample t-test are performed using untransformed outcomes for comparative purposes.
3. Steps 1–2 are repeated *N*=100000 times and the power of the corresponding hypothesis test is calculated as the proportion of these repeated tests for which the null hypothesis is rejected.

## Results

Table 1 shows results of the simulation study for various pre-specified median outcome values and standard deviations. The column ‘*n*’ denotes the analytically derived sample size calculated using Eq. (4). The columns ‘log t-test’, ‘M-W test ’ and ‘t-test’ denote the estimated hypothesis test powers for the t-test on log-transformed outcomes, the Mann-Whitney U test on untransformed outcomes and the t-test on untransformed outcomes, respectively, between groups. Examining Table 1, for larger sample sizes the two-sample t-test of the log-transformed outcomes between groups appears to have a power close to the nominal power value (either 0.8 or 0.9). For smaller sample sizes the power is sometimes slightly less than the nominal value. In such situations, it would be advisable to increase the sample size slightly, perhaps by one or two individuals in each group, to ensure that the desired level of power is attained. This issue is likely to be caused by the fact that the sample size calculation in Eq. (4) uses normal distribution quantiles $z_{\frac {\alpha }{2}}, z_{\beta }$ but the hypothesis test that is performed is a two-sample t-test. This would also affect any sample size calculation where a normal approximation is used and the sample size is small and is not specific to the approach taken in this work.

**Table 1:** Simulation study results

Significance level = 0.05, Power = 0.8							
					Estimated power from simulation study		
*m*_1_	*m*_2_	*ϕ*_1_	*ϕ*_2_	*n*	log t-test	M-W test	t-test
1	1.5	0.5	0.5	14	0.781	0.755	0.690
1	1.25	0.5	0.5	51	0.797	0.776	0.664
1	1.1	0.5	0.5	303	0.801	0.781	0.639
1	0.5	0.4	0.4	9	0.788	0.724	0.687
1	0.7	0.4	0.4	23	0.794	0.772	0.662
1	0.9	0.4	0.4	204	0.800	0.781	0.669
1	0.6	0.3	0.3	9	0.791	0.728	0.728
1	0.7	0.3	0.3	15	0.800	0.762	0.723
1	0.8	0.3	0.3	32	0.797	0.773	0.713
1	0.75	0.25	0.25	15	0.784	0.747	0.729
1	0.88	0.25	0.25	63	0.797	0.776	0.737
1	0.94	0.25	0.25	250	0.800	0.781	0.741
Significance level = 0.05, Power = 0.9							
					Estimated power from simulation study		
*m*_1_	*m*_2_	*ϕ*_1_	*ϕ*_2_	*n*	log t-test	M-W test	t-test
1	1.5	0.5	0.7	23	0.888	0.873	0.847
1	1.25	0.5	0.7	87	0.898	0.883	0.910
1	1.1	0.5	0.7	530	0.900	0.886	0.992
1	0.5	0.6	0.4	14	0.890	0.872	0.800
1	0.7	0.6	0.4	40	0.897	0.882	0.875
1	0.9	0.6	0.4	383	0.900	0.886	0.993
1	0.6	0.5	0.3	16	0.896	0.874	0.867
1	0.7	0.5	0.3	28	0.894	0.877	0.897
1	0.8	0.5	0.3	65	0.896	0.880	0.946
1	0.75	0.4	0.25	29	0.892	0.875	0.906
1	0.88	0.4	0.25	131	0.898	0.882	0.970
1	0.94	0.4	0.25	537	0.900	0.883	0.998

Here, *m*_1_,*m*_2_ are pre-specified untransformed median values for groups 1 and 2, with *ϕ*_1_,*ϕ*_2_ corresponding untransformed standard deviations. The column ‘*n*’ denotes the analytically derived sample size calculated using Eq. (4). Estimated powers are shown for a two-sample t-test of log-transformed outcomes (‘log t-test’), a Mann-Whitney U test of untransformed outcomes (‘M-W test’) and a two-sample t-test of untransformed outcomes (‘t-test’)

The ‘M-W Test’ column shows that a Mann-Whitney U test on untransformed outcomes does not attain the expected power in all situations, being smaller than the desired level of power in each case. We would expect this and, consequently, suggest a suitable adjustment to the sample size calculation if a Mann-Whitney U test or other non-parametric hypothesis test were used [9, 10]. Furthermore, we note that the Mann-Whitney U test may be viewed as a test of a shift in location of the outcome variable’s probability distribution between groups. As such, it may not be desirable to consider a Mann-Whitney U test in situations where the untransformed variances differ between groups. However, in Table 1 estimated powers from the Mann-Whitney U test are not typically worse for simulation scenarios where *ϕ*_1_≠*ϕ*_2_ when compared to scenarios where *ϕ*_1_=*ϕ*_2_. Hence, the issue of different variances between groups does not appear to have been too problematic here.

When considering the ‘t-test’ column of Table 1, where the untransformed standard deviation values are equal for groups 1 and 2 (*ϕ*_1_=*ϕ*_2_), a two-sample t-test of untransformed outcomes consistently fails to attain the desired level of power, even when the sample size is large. However, where untransformed standard deviation values are different (*ϕ*_1_≠*ϕ*_2_) we see that there are some scenarios where a power greater than the pre-specified power is attained. We note that the two-sample t-test tests the null hypothesis that the untransformed population mean values are equal. Here, for untransformed log-normal outcomes, the population mean for group *j* is given by $m_{j}\exp \left ({\sigma ^{2}_{j}}/2\right)$ and hence if *m*_1_=*m*_2_ but $\phi _{1}\neq \phi _{2}{\left (\text {implying that}~\sigma _{1}^{2} \neq \sigma _{2}^{2}\right)}$, the null hypothesis for a two-sample t-test of untransformed outcomes would never be true. This explains why the estimated power can be considerably higher than the pre-specified value in this situation. Naturally, we would not usually recommend a two-sample t-test on untransformed outcomes where the data have a log-normal or other positively skewed probability distribution and, in most cases, a histogram of outcome data would indicate that a suitable transformation is required prior to using a t-test.

Overall, the results in Table 1 indicate that the analytical method given in Eq. (4) appears to give correct sample sizes for desired hypothesis test powers based on median values and untransformed standard deviations for outcomes of interest. Some adjustment may be necessary for smaller sample sizes but such adjustment would be recommended with most sample size calculation methods.

### Sensitivity to the log-normal distributional assumption

**Fig. 1:**
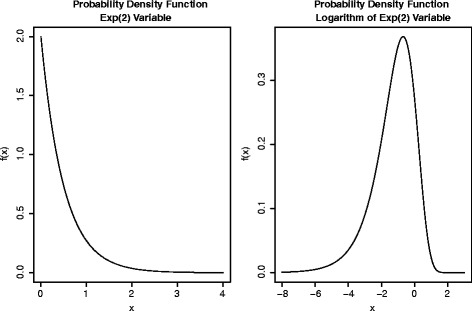
Plots showing the probability density function of an Exp(2) random variable (left-hand plot) and the natural logarithm of an Exp(2) random variable (right-hand plot)

When health outcomes have a positively skewed distribution, the distribution may not be strictly log-normal. Here, we simulate some scenarios to consider the performance of the sample size method where the distribution is skewed but not log-normal. Specifically, we examine situations where the outcome has an Exponential distribution. We note that it can be easily shown that the logarithm of an Exponential random variable is not normally distributed. As an example, Fig. 1 shows probability density functions of an Exp(2) random variable and the natural logarithm of an Exp(2) random variable. If a random variable *X*∼Exp(*λ*) then the median of *X* is equal to log(2)/*λ* and the standard deviation is 1/*λ*. The fact that a closed form expression for the median exists is desirable here, since our sample size calculation method (Eq. (4)) relies on pre-specified median values that, with an Exponential distribution, can be directly linked to rate parameters. We perform sample size calculations using Eq. (4) for pre-specified median and untransformed standard deviation values under a log-normal assumption, but then simulate data from Exponential distributions with the same median and standard deviation values. The simulation algorithm is similar to that outlined previously, except that Exponential distribution rates for groups are calculated as *λ*_*j*_= log(2)/*m*_*j*_ (where *m*_*j*_ is the pre-specified median for group *j*) and then untransformed values are drawn from an Exp(*λ*_*j*_) distribution for group *j*. This simulation process allows the evaluation of the sample size calculation method where the outcome distribution is not strictly log-normal in that we can assess the expected level of power when performing various hypothesis tests.

**Fig. 2:**
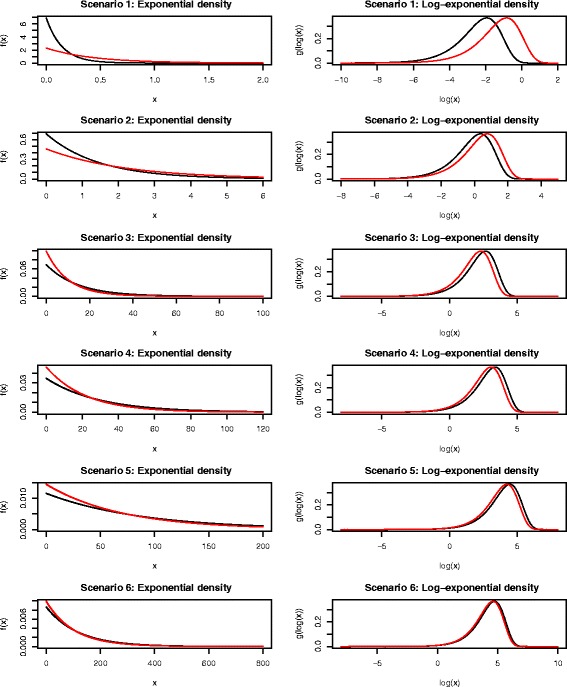
Plots showing the probability density functions of the Exponential distribution (left-hand column) and log-Exponential distribution (right-hand column) for each simulation scenario of Table 2. Black lines indicate densities for group 1 and the red lines those for group 2

Results from the simulation study where the outcome data have an Exponential distribution are shown in Table 2. Each row of Table 2 denotes a different simulation scenario and Fig. 2 shows example plots of the distributions of values and log-transformed values for each scenario to demonstrate levels of skewness. Figure 2 shows that the log-transformed outcomes generally exhibit left skewness.

**Table 2:** Results from the simulation study where outcome data have Exponential distributions

Significance level = 0.05, Power = 0.9								
						Estimated power from simulation study		
Scenario	*m*_1_	*m*_2_	*ϕ*_1_	*ϕ*_2_	*n*	log t-test	M-W test	t-test
1	0.1	0.3	$\frac {0.1}{\log (2)}$	$\frac {0.3}{\log (2)}$	13	0.576	0.600	0.661
2	1	1.5	$\frac {1}{\log (2)}$	$\frac {1.5}{\log (2)}$	91	0.567	0.650	0.769
3	10	7	$\frac {10}{\log (2)}$	$\frac {7}{\log (2)}$	117	0.564	0.649	0.768
4	20	15	$\frac {20}{\log (2)}$	$\frac {15}{\log (2)}$	180	0.565	0.654	0.772
5	60	48	$\frac {60}{\log (2)}$	$\frac {48}{\log (2)}$	299	0.564	0.653	0.775
6	80	70	$\frac {80}{\log (2)}$	$\frac {70}{\log (2)}$	833	0.565	0.654	0.778

Here, *m*_*j*_ and *ϕ*_*j*_ denote the median and standard deviation of the outcome data for group *j*. Estimated powers are shown for a two-sample t-test of log-transformed outcomes (‘log t-test’), a Mann-Whitney U test of untransformed outcomes (‘M-W test’) and a two-sample t-test of untransformed outcomes (‘t-test’)

Examining Table 2 we see that the estimated power for each method is below the nominal value of 0.9 for all scenarios. On examining the form of Eq. (4) and the untransformed variances for the Exponential distribution, the poor performance of the methods is unsurprising. The standard deviations used in the sample size calculation in Eq. (4) are too small to reflect the true variability of the log-transformed exponentially distributed data, resulting in sample size calculations that are too small to attain the deired level of power under the proposed method. For the Exponential distributions, the untransformed variance in group *j* is given by

$$\phi_{j}^{2} = \frac{m_{j}^{2}}{[\log(2)]^{2}} $$

and, therefore, on substitution into the formula for the log-transformed variance in Eq. 4 we obtain

$$\begin{array}{*{20}l} \sigma_{j}^{2} &= \log\left(\frac{1}{2}+\sqrt{\frac{1}{4}+\frac{1}{m_{j}^{2}}\frac{m_{j}^{2}}{[\log(2)]^{2}}} \right)\\ &= \log\left(\frac{1}{2}+\sqrt{\frac{1}{4}+\frac{1}{[\log(2)]^{2}}}\right)\\ &\approx 0.7065. \end{array} $$

for all *j*. It can be shown that the variance of the natural logarithm of an Exponential random variable is *π* ^2^/6≈1.645 (see ‘Additional file 1’). This is more than twice the assumed value of $\sigma _{j}^{2}$ that was used for sample size calculations given in Table 2 which explains why the estimated powers in Table 2 are low. To amend the sample size calculation in Eq. (4) for this distribution, we substitute $\sigma _{j}^{2} = \pi ^{2}/6$ into Eq. (4) and re-calculate the sample sizes using the formula

5 $$ n = \frac{\left(\pi^{2}/6+\pi^{2}/6\right)\left(z_{\frac{\alpha}{2}}+z_{\beta}\right)^{2}}{(\log(m_{1})-\log(m_{2}))^{2}}.  $$

Table 3 shows results from a simulation study, conducted in the same way as that in Table 2, but where the analytical sample sizes have been calculated using Eq. (5). We see that calculation of the sample size using Eq. (5) results in larger sample size values, which we would expect as more accurate estimates of the transformed variance values have been used. As a result, a power close to 0.9 is attained for all simulation scenarios in which a two-sample t-test is performed on the log-transformed outcomes. However, the power is typically higher for the Mann-Whitney U test, thereby suggesting that this test would generally require a smaller sample size than the two-sample t-test on the log-transformed outcomes. This might be expected, since we can see in Fig. 2 that the log-transformed outcomes are negatively skewed and thus the data are generally more suited to analysis using a Mann-Whitney U test. Also, we note that in Table 3 the estimated powers for the t-test using untransformed outcomes are very high. Naturally, this test would not typically be used for exponentially distributed outcome data. In summary, for positively skewed outcomes that are clearly not log-normal, the sample size calculation presented in Eq. (4) should not be used without carefully considering the shape of the distribution of log-transformed outcomes and its variability.

**Table 3:** Results from the simulation study where outcome data have Exponential distributions, with analytic sample sizes (*n*) calculated using the formula given in Eq. 5

Significance level = 0.05, Power = 0.9								
						Estimated power from simulation study		
Scenario	*m*_1_	*m*_2_	*ϕ*_1_	*ϕ*_2_	*n*	log t-test	M-W test	t-test
1	0.1	0.3	$\frac {0.1}{\log (2)}$	$\frac {0.3}{\log (2)}$	29	0.890	0.933	0.975
2	1	1.5	$\frac {1}{\log (2)}$	$\frac {1.5}{\log (2)}$	211	0.900	0.948	0.985
3	10	7	$\frac {10}{\log (2)}$	$\frac {7}{\log (2)}$	272	0.898	0.947	0.985
4	20	15	$\frac {20}{\log (2)}$	$\frac {15}{\log (2)}$	418	0.900	0.950	0.986
5	60	48	$\frac {60}{\log (2)}$	$\frac {48}{\log (2)}$	695	0.899	0.949	0.985
6	80	70	$\frac {80}{\log (2)}$	$\frac {70}{\log (2)}$	1939	0.898	0.949	0.985

Here, *m*_*j*_ and *ϕ*_*j*_ denote the median and standard deviation of the outcome data for group *j*. Estimated powers are shown for a two-sample t-test of log-transformed outcomes (‘log t-test’), a Mann-Whitney U test of untransformed outcomes (‘M-W test’) and a two-sample t-test of untransformed outcomes (‘t-test’)

We now present a real example in which the analytical method has been used to obtain a sample size for a two arm randomised controlled trial in epilepsy surgery for which the outcome of interest is the time taken for the implantation of electrodes during a surgical procedure.

## Application

Some patients with refractory focal epilepsy, a type of epilepsy that is difficult to control using medication alone, may be considered for neurosurgery whereby invasive testing is required to determine the location of epileptic activity in the brain [14]. This procedure is known as stereoelectroencephalography (SEEG) and involves the implantation of SEEG electrodes into a patient’s brain in a surgical procedure. These electrodes are monitored using continual video and encephalography monitoring to assess brain activity. A number of different methods exist for the placement of SEEG electrodes and it is unclear which placement method is best [15].

A randomised controlled trial of SEEG electrode placement methods has been funded at the National Hospital for Neurology and Neurosurgery, London (Wellcome Trust grant number WT106882), in which the operative time (time taken for electrode implantation procedure) of the iSYS1 trajectory guidance system is to be compared with the currently used frameless mechanical arm based technique for the placement of SEEG depth electrode bolts in patients undergoing pre-operative evaluation for drug resistant focal epilepsy. In brief, the iSYS1 trajectory guidance system [16] uses a robot during part of the surgical procedure for the implantation of the electrodes into a patient’s brain. It is believed that the robot insertion method will result in a significant reduction in the time taken to perform SEEG electrode placement [16, 17].

For this randomised controlled trial, the primary outcome is the time taken (in minutes) for the implantation of an electrode during surgery. Typically, each patient has 8–12 electrodes implanted during a surgical procedure and, as such, we note that a degree of patient-level clustering is to be expected with regard to the primary outcome in this example. Patients shall be randomised to one of two groups, with equal allocation, and the two randomised groups are:

- Group 1: Patients who are randomised to receive manual SEEG electrode placement;
- Group 2: Patients who are randomised to receive robot-guided SEEG electrode placement.

The trial investigators aimed to estimate the number of patients to recruit to the trial so that a reduction of at least 20% in the median electrode implantation time may be detected when comparing times for electrode implantation between groups. Clearly, implantation times are likely to be positively skewed and, as such, analysis of the primary outcome for this trial shall consist of a two-sample t-test of the null hypothesis of no difference in electrode implantation time between groups, by comparing log-transformed implantation times. A 5% significance level and a power of 90% are assumed. For this trial, we first perform a sample size calculation using Eq. (4) where the median electrode implantation time for manual SEEG electrode placement (*m*_1_) is specified, together with estimates of the standard deviation of the implantation time for an electrode for both groups (*ϕ*_1_,*ϕ*_2_). The values assumed for the sample size calculation are:

$$\begin{array}{*{20}l} m_{1} &= 20\text{~min};\\ m_{2} &= 0.8\times20 = 16 \text{~min, since there is an }\\ &\qquad\text{anticipated 20\% reduction.};\\ \phi_{1} &= 5 \text{~min};\\ \phi_{2} &= 5 \text{~min};\\ z_{\frac{\alpha}{2}} &= 1.96 \text{~(5\% significance level)};\\ z_{\beta} &= 1.28 \text{~(90\% power)}. \end{array} $$

Ignoring the clustering for now, we use Eq. (4) to compute the number of electrodes required in each arm as

$$\begin{aligned} n &= \frac{\left[\log\left(\frac{1}{2}+\sqrt{\frac{1}{4}+\frac{5^{2}}{20^{2}}}\right)+\log\left(\frac{1}{2}+\sqrt{\frac{1}{4}+\frac{5^{2}}{16^{2}}}\right)\right](1.96+1.28)^{2}}{(\log(20)-\log(16))^{2}}\\ &= 30.18. \end{aligned} $$

This would equate to a sample size of 31 electrodes per group. Since this sample size is fairly small and, in light of the simulation study results, we increase the sample size to 32 electrodes per group. However, we note that electrodes are clustered within patients who undergo surgery and, as such, the effect of within-patient clustering should be accounted-for in the sample size calculation. Clustering can be handled easily within our sample size calculation approach.

### Accounting for clustering

In a similar way to other sample size calculation methods, we can calculate a design effect that is a function the intra-class correlation coefficient and the average cluster size and use this to update the sample size calculation to account for the likely effect of within-patient clustering [13]. The design effect is calculated as:

$$\text{def} = 1+\text{ICC}(m-1) $$

where *m*=10 is the average cluster size and ICC = 0.2 is a estimated value for the intra-class correlation coefficient. Here, def=2.8 and the original sample size is inflated by this factor to reflect the within-patient clustering [18], yielding a revised sample size of

$$\begin{array}{*{20}l} n^{*} &= 2.8\times32\\ &= 89.6. \end{array} $$

This implies that each group should contain 90 electrodes and this would suggest that 9 patients per group should be recruited to trial, under the assumption of an average of 10 electrode insertions per patient. In the protocol for the SEEG Electrode Placement Randomised Controlled Trial, the number of patients to be recruited has been increased to 16 per group, to reflect the possibility of patient drop-out and variable cluster size.

## Discussion

We have considered a sample size calculation method in which clinical efficacy is measured using median values for a positively skewed outcome that is assumed to follow a log-normal distribution. We chose this approach because, particularly in the case of times and other positively skewed health outcomes, comparisons between groups may be specified based on differences in medians (or, equivalently, differences in geometric means) rather than differences in arithmetic means. Furthermore, information on variability of the untransformed outcome variable may be easier to estimate and more interpretable than that of a transformed outcome variable. In addition, the approach is applicable to situations in which interest lies in a direct comparison of the log-scale means between groups or for situations in which linear models are fitted to log-transformed outcome data. The example on SEEG electrode placement showed that this approach to sample size calculation could be applicable in clinical practice and also that clustering of outcome data can be handled within the outlined sample size method.

The analytical sample size formula presented (Eq. (4)) was accurate when evaluated using extensive simulation studies. As such, the method appears to be acceptable for estimation of the required sample size for situations in which two groups are compared and the outcome of interest is assumed to have a log-normal distribution. We note that the assumption of a log-normal distribution may not always be appropriate. A simulation study in which outcomes had an exponential distribution indicated that the formula in Eq. (4) provided sample sizes that were generally too small. However, an adjustment to Eq. (4) that used a more precise estimate of the untransformed variance yielded a formula for which the approach outlined appeared to work well. In general, we recommend that caution should be taken when using the approach presented in this paper if it is suspected that outcomes are not log-normal. In such situations we would recommend simulation studies to check that the proposed analytical sample size method is effective. Alternatively, if a different distributional assumption is made for untransformed outcomes, such as a Gamma distribution, then a likelihood ratio test statistic could be constructed based on that distributional assumption and used to calculate a sample size [19]. In some cases, it may be more appropriate to consider a non-parametric hypothesis test of untransformed outcomes, though we note that a parametric test or other analysis that relies on the specification of a probability distribution for outcome variables may be useful as a sensitivity analysis.

Additionally, as with all sample size approaches, the method depends on the pre-specified values (i.e. the estimated medians and standard deviations of the untransformed outcomes). As a result, it is important to elicit pre-specified values that are applicable to the study at hand. Here, we note that it may be more appropriate and perhaps easier for health care researchers to specify median values and associated standard deviations of the untransformed (clinically interpretable) outcome variable than to consider estimated values for log-transformed outcomes. Furthermore, the median may represent a more robust and intuitive summary of an outcome for a treatment group where the distribution is positively skewed, when compared to the sample mean which may be affected by extreme values.

The sample size calculations were performed on the assumption of equal numbers of individuals in the two groups. The calculations could be easily adapted to situations in which the numbers in the two groups are unequal, using standard adjustment methods. Overall, we have presented a simple method for sample size calculation where the outcome of interest is assumed to have a log-normal distribution and a hypothesis test is performed using data from two groups. The method is applicable to problems in which a difference in median values between the groups is of clinical interest and untransformed standard deviations are specified.

## Conclusions

The sample size approach that has been outlined is applicable to situations in which a comparison between medians or log-transformed means is proposed for positively skewed data under the assumption that the data have a log-normal distribution. The method relies on pre-specified untransformed median and standard deviation values for groups which may typically be easier to elicit from clinicians and perhaps more interpretable. The method may be adjusted to account for situations where the outcome data are positively skewed but not strictly log-normal.

## Additional file

Additional file 1 Properties of the log-normal distribution, derivation of the variance of the log-Exponential distribution and simulation study R code. Derivation of the relationship between the median of a log-normal random variable and the mean of its log-transform. Derivation of the form of the variance of the log-transformed outcome and that for the log-exponential distribution. R code for the simulation studies presented in this paper. (PDF 164 kb)

## Abbreviations

ICC: Intra-class correlation coefficient
SEEG: Stereoelectroencephalography
