# Supplementary material for: Sample size calculations based on a difference in medians for positively skewed outcomes in health care studies
Source: BMC Med Res Methodol. 2017 Dec 2;17:157. doi: 10.1186/s12874-017-0426-1 (PMC5712177; doi:10.1186/s12874-017-0426-1)
Supplement: Additional file 1 — Properties of the log-normal distribution, derivation of the variance of the log-Exponential distribution and simulation study R code. Derivation of the relationship between the median of a log-normal random variable and the mean of its log-transform. Derivation of the form of the variance of the log-transformed outcome and that for the log-exponential distribution. R code for the simulation studies presented in this paper. (PDF 164 kb) [file 12874_2017_426_MOESM1_ESM.pdf]

Additional File 1: Properties of the log-normal distribution, derivation of the variance of the log-Exponential distribution and simulation study R code

Aidan G. O'Keeffe[*], Gareth Ambler, and Julie A. Barber

Department of Statistical Science, University College London, WC1E 6BT, UK

Relationship between the median of a log-normal random variable and the mean of its log-transform

Let

$$T_{ij} \sim \log\mathcal{N}(\mu_j, \sigma_j^2).$$

Defining m_j as the median of the probability distribution of T_{ij}, m_j is the value such that

$$\mathbb{P}(T_{ij} \leq m_j) = \frac{1}{2}.$$

Since the natural logarithm is a monotonic increasing function of T_{ij}, it follows that

$$\mathbb{P}(\log(T_{ij}) \leq \log(m_j)) = \frac{1}{2}$$

Recalling that $\log(T_{ij}) \sim \mathcal{N}(\mu_j, \sigma_j^2)$, then

$$Z_{ij} = \frac{\log(T_{ij}) - \mu_j}{\sigma_j} \sim \mathcal{N}(0, 1).$$

[*]E-mail address: a.o'keeffe@ucl.ac.uk; Corresponding author

Thus,

$$\begin{aligned} &\mathbb{P}(\log(T_{ij}) \leq \log(m_j)) = \frac{1}{2}; \\ \implies &\mathbb{P}\left(\frac{\log(T_{ij}) - \mu_j}{\sigma_j} \leq \frac{\log(m_j) - \mu_j}{\sigma_j}\right) = \frac{1}{2}; \\ \implies &\mathbb{P}\left(Z_{ij} \leq \frac{\log(m_j) - \mu_j}{\sigma_j}\right) = \frac{1}{2}. \end{aligned}$$

Hence,

$$\frac{\log(m_j) - \mu_j}{\sigma_j} = \Phi^{-1}\left(\frac{1}{2}\right) = 0$$

where $\Phi^{-1}()$ denotes the inverse cumulative density function of a standard $\mathcal{N}(0, 1)$ random variable. As a result

$$\begin{aligned} &\log(m_j) - \mu_j = 0 \\ &\qquad \implies m_j = \exp(\mu_j), \text{ as required.} \end{aligned}$$

Derivation of variance of log-transformed outcome

Let

$$T_{ij} \sim \log\mathcal{N}(\mu_j, \sigma_j^2).$$

Then

$$\text{Var}(T_{ij}) = \left(\exp(\sigma_j^2) - 1\right) \exp\left(2\mu_j + \sigma_j^2\right) = \phi_j^2.$$

It follows that

$$
\begin{aligned}
\big(\exp(\sigma_j^2) - 1\big)\exp(\sigma_j^2)\exp(2\mu_j) &= \phi_j^2 \\
\exp(2\mu_j)\exp(2\sigma_j^2) - \exp(2\mu_j)\exp(\sigma_j^2) - \phi_j^2 &= 0 \\
\exp(2\sigma_j^2) - \exp(\sigma_j^2) - \frac{\phi_j^2}{\exp(2\mu_j)} &= 0 \\
\left(\exp(\sigma_j^2) - \frac{1}{2}\right)^2 - \frac{1}{4} - \frac{\phi_j^2}{\exp(2\mu_j)} &= 0 \\
\left(\exp(\sigma_j^2) - \frac{1}{2}\right)^2 &= \frac{1}{4} + \frac{\phi_j^2}{\exp(2\mu_j)} \\
\exp(\sigma_j^2) - \frac{1}{2} &= \sqrt{\frac{1}{4} + \frac{\phi_j^2}{\exp(2\mu_j)}} \\
\exp(\sigma_j^2) &= \frac{1}{2} + \sqrt{\frac{1}{4} + \frac{\phi_j^2}{\exp(2\mu_j)}} \\
\sigma_j^2 &= \log\left[\frac{1}{2} + \sqrt{\frac{1}{4} + \frac{\phi_j^2}{\exp(2\mu_j)}}\right] \text{ as required.}
\end{aligned}
$$

Derivation of Variance of Logarithm of an Exponential Random Variable

Let $X \sim \text{Exp}(\lambda)$, then

$$f_X(x; \lambda) = \lambda \exp(-\lambda x) \quad (x > 0)$$

Consider $\mathbb{E}(\log(X))$:

$$
\begin{aligned}
\mathbb{E}(\log(X)) &= \int_0^\infty \log(x)\lambda\exp(-\lambda x)\mathrm{d}x \\
&= \int_0^\infty [\log(y) - \log(\lambda)]\exp(-y)\mathrm{d}y \quad \text{(using the substitution } y = \lambda x\text{)} \\
&= \gamma - \log(\lambda)
\end{aligned}
$$

where γ is the Euler-Mascheroni constant

$$\gamma = \int_0^\infty \log(y)\exp(-y)\mathrm{d}y.$$

The variance of $\log(X)$ is given by

$$\mathrm{Var}(\log(X)) = \mathbb{E}[(\log(X))^2] - \mathbb{E}(\log(X))^2 \tag{1}$$

Consider $\mathbb{E}\left[(\log(X))^2\right]$:

$$\begin{aligned}
\mathbb{E}\left[(\log(X))^2\right] &= \int_0^\infty (\log(x))^2 \lambda \exp(-\lambda x)\mathrm{d}x \\
&= \int_0^\infty (\log(y) - \log(\lambda))^2 e^{-y}\mathrm{d}y \\
&= \int_0^\infty (\log(y))^2 e^{-y}\mathrm{d}y - 2\log(\lambda)\int_0^\infty \log(y)e^{-y}\mathrm{d}y + (\log(\lambda))^2 \int_0^\infty e^{-y}\mathrm{d}y \\
&= \frac{\pi^2}{6} + \gamma^2 - 2\left[\gamma - \log(\lambda)\right] + (\log(\lambda))^2 \\
&= \frac{\pi^2}{6} + (\log(\lambda) - \gamma)^2
\end{aligned}$$

and substitution of the above into (1) yields

$$\begin{aligned}
\mathrm{Var}(\log(X)) &= \frac{\pi^2}{6} + (\log(\lambda) - \gamma)^2 - (\gamma - \log(\lambda))^2 \\
&= \frac{\pi^2}{6} \quad \text{as required.}
\end{aligned}$$

R Code for the Simulation Study Presented in Table 1

The function '`logn_sim_study`' simulates datasets of a pre-specified size (n) drawn from log-normal distributions with pre-specified medians m_1, m_2 and standard deviations ϕ_1 and ϕ_2 for groups 1 and 2. Simulated data for the groups are then compared using: (1) a two-sample t-test on log-transformed outcomes, (2) A Mann-Whitney U test on untransformed outcomes, (3) A two-sample t-test on untransformed outcomes. This code is repeated 100000 times, for each scenario, for the simulation study presented in Table 1 of the paper.

```
#Argument 'pars' is a 5x1 vector of parameters where
#pars[1] = Pre-specified median for group 1
```

```
#pars[2] = Pre-specified median for group 2
#pars[3] = Pre-specified standard deviation (untransformed) for group 1
#pars[4] = Pre-specified standard deviation (untransformed) for group 2
#pars[5] = n = Analytical sample size (calculated using Equation (4) from the paper).

logn_sim_study<-function(pars){
 median1<-pars[1]
 median2<-pars[2]
 phi1<-pars[3]
 phi2<-pars[4]
 n<-pars[5]

#Recover log-scale means using the pre-specified medians
 mu1<-log(median1)
 mu2<-log(median2)

#Recover standard deviations on the log-scale using the pre-specified medians,
#untransformed variances and the Equation (2) from the paper.
 sigma1<-sqrt(log(1/2+sqrt(1/4+((phi1^2)/(median1^2)))))
 sigma2<-sqrt(log(1/2+sqrt(1/4+((phi2^2)/(median2^2)))))

#Simulate n values from log-normal distributions for each of groups 1 and 2.
#For group 1: log-scale mean = mu1, standard deviation of
#log-transformed outcomes = sigma1;
#For group 2: log-scale mean = mu2, standard deviation of
#log-transformed outcomes = sigma2.

 y1<-rlnorm(n,meanlog=mu1,sdlog=sigma1)
 y2<-rlnorm(n,meanlog=mu2,sdlog=sigma2)

#Perform a two-sample t-test on log-transformed outcomes.
 log_ttest<-t.test(log(y1),log(y2),var.equal=TRUE)

#Extract the P-value from the two-sample t-test on log-transformed outcomes.
 logn_pval<-log_ttest$p.value
```

```
#Create a binary variable that equals 1 if the null hypothesis is rejected
#and 0 otherwise.
 logn_reject<-ifelse(logn_pval<0.05,1,0)

#Perform a Mann-Whitney U test on untransformed outcomes.
 mw_test<-wilcox.test(y1,y2,alternative="two.sided")

#Extract the P-value from the Mann-Whitney U test on untransformed outcomes.
 mw_pval<-mw_test$p.value

#Create a binary variable that equals 1 if the null hypothesis is rejected
#and 0 otherwise.
 mw_reject<-ifelse(mw_pval<0.05,1,0)


#Perform a two-sample t-test on untransformed outcomes.
 ttest<-t.test(y1,y2,var.equal=TRUE)

#Extract the P-value from the two-sample t-test on log-transformed outcomes.
 t_pval<-ttest$p.value

#Create a binary variable that equals 1 if the null hypothesis is rejected
#and 0 otherwise.
 t_reject<-ifelse(t_pval<0.05,1,0)

#Produce a table of results and output this.
 results<-data.frame(logn_pval,logn_reject,mw_pval,mw_reject,t_pval,t_reject)
 names(results)<-c("log_N_P_Value","log_N_Reject","MW_P_Value","MW_Reject",
                   "t_test_P_Value","t_test_Reject")
 return(results)
 }
```

R Code for the Simulation Studies Presented in Tables 2 and 3

The function '`exponential_sim_study`' simulates datasets of a pre-specified size (n) drawn from Exponential distributions with pre-specified rates $\lambda_1 = \log(2)/m_1$, $\lambda_2 = \log(2)/m_2$ for groups 1 and 2 respectively. Simulated data for the groups are then compared using: (1) a two-sample t-test on log-transformed outcomes, (2) A Mann-Whitney U test on untransformed outcomes, (3) A two-sample t-test on untransformed outcomes. This code is repeated 100000 times, for each senario, for the simulation study presented in Tables 2 and 3 of the paper.

```
#Argument 'pars' is a 3x1 vector of parameters where
#pars[1] = Pre-specified median for group 1
#pars[2] = Pre-specified median for group 2
#pars[3] = n = Analytical sample size (calculated using Equation (4) from
#the paper for simulation study in Table 2, calculated using Equation (5)
#from the paper for simulation study in Table 3).

exponential_sim_study<-function(pars){

 median1<-pars[1]
 median2<-pars[2]
 n<-pars[3]

#Compute the rates 'lambda1' for group 1 and 'lambda2' for group 2.
 lambda1<-log(2)/median1
 lambda2<-log(2)/median2

#Simulate n values from Exponential distributions for each of groups 1 and 2.
#y1 = simulated values for group 1;
#y2 = simualted values for group 2.

 y1<-rexp(n,rate=lambda1)
 y2<-rexp(n,rate=lambda2)

#Take log-transforms of simulated outcomes for groups 1 and 2, denoting
```

```
#these 'logy1' and 'logy2' respectively.
 logy1<-log(y1)
 logy2<-log(y2)

#Perform a two-sample t-test on log-transformed outcomes.
 log_ttest<-t.test(log(y1),log(y2),var.equal=TRUE)

#Extract the P-value from the two-sample t-test on log-transformed outcomes.
 logn_pval<-log_ttest$p.value

#Create a binary variable that equals 1 if the null hypothesis is rejected
#and 0 otherwise.
 logn_reject<-ifelse(logn_pval<0.05,1,0)


#Perform a Mann-Whitney U test on untransformed outcomes.
 mw_test<-wilcox.test(y1,y2,alternative="two.sided")

#Extract the P-value from the Mann-Whitney U test on untransformed outcomes.
 mw_pval<-mw_test$p.value

#Create a binary variable that equals 1 if the null hypothesis is rejected
#and 0 otherwise.
 mw_reject<-ifelse(mw_pval<0.05,1,0)

#Perform a two-sample t-test on untransformed outcomes.
 ttest<-t.test(y1,y2,var.equal=TRUE)

#Extract the P-value from the two-sample t-test on log-transformed outcomes.
 t_pval<-ttest$p.value

#Create a binary variable that equals 1 if the null hypothesis is rejected
#and 0 otherwise.
 t_reject<-ifelse(t_pval<0.05,1,0)

#Produce a table of results and output this.
```

```
results<-data.frame(logn_pval,logn_reject,mw_pval,mw_reject,t_pval,t_reject)
names(results)<-c("log_N_P_Value","log_N_Reject","MW_P_Value","MW_Reject",
                  "t_test_P_Value","t_test_Reject")
return(results)
}
```
